# Large-Scale Discovery of Promoter Motifs in Drosophila melanogaster

**DOI:** 10.1371/journal.pcbi.0030007

**Published:** 2007-01-19

**Authors:** Thomas A Down, Casey M Bergman, Jing Su, Tim J. P Hubbard

**Affiliations:** 1 Wellcome Trust Sanger Institute, Hinxton, Cambridge, United Kingdom; 2 Faculty of Life Sciences, University of Manchester, Manchester, United Kingdom; Duke University, United States of America

## Abstract

A key step in understanding gene regulation is to identify the repertoire of transcription factor binding motifs (TFBMs) that form the building blocks of promoters and other regulatory elements. Identifying these experimentally is very laborious, and the number of TFBMs discovered remains relatively small, especially when compared with the hundreds of transcription factor genes predicted in metazoan genomes. We have used a recently developed statistical motif discovery approach, NestedMICA, to detect candidate TFBMs from a large set of Drosophila melanogaster promoter regions. Of the 120 motifs inferred in our initial analysis, 25 were statistically significant matches to previously reported motifs, while 87 appeared to be novel. Analysis of sequence conservation and motif positioning suggested that the great majority of these discovered motifs are predictive of functional elements in the genome. Many motifs showed associations with specific patterns of gene expression in the D. melanogaster embryo, and we were able to obtain confident annotation of expression patterns for 25 of our motifs, including eight of the novel motifs. The motifs are available through Tiffin, a new database of DNA sequence motifs. We have discovered many new motifs that are overrepresented in D. melanogaster promoter regions, and offer several independent lines of evidence that these are novel TFBMs. Our motif dictionary provides a solid foundation for further investigation of regulatory elements in *Drosophila,* and demonstrates techniques that should be applicable in other species. We suggest that further improvements in computational motif discovery should narrow the gap between the set of known motifs and the total number of transcription factors in metazoan genomes.

## Author Summary

In contrast to the genomic sequences that encode proteins, little is known about the regulatory elements that instruct the cell as to when and where a given gene should be active. Regulatory elements are thought to consist of clusters of short DNA words (motifs), each of which acts as a binding site for sequence-specific DNA binding protein. Thus, building a comprehensive dictionary of such motifs is an important step towards a broader understanding of gene regulation. Using the recently published NestedMICA method for detecting overrepresented motifs in a set of sequences, we build a dictionary of 120 motifs from regulatory sequences in the fruitfly genome, 87 of which are novel. Analysis of positional biases, conservation across species, and association with specific patterns of gene expression in fruitfly embryos suggest that the great majority of these newly discovered motifs represent functional regulatory elements. In addition to providing an initial motif dictionary for one of the most intensively studied model organisms, this work provides an analytical framework for the comprehensive discovery of regulatory motifs in complex animal genomes.

## Introduction

Essentially complete sequences of metazoan genomes have now been available for nearly ten years, and in that time considerable progress has been made towards annotation of their best-known features, the protein coding genes. Computational pipelines such as that run by Ensembl [1] provide automated annotation of most protein-coding genes, which for some genomes have been augmented by manual curation to improve the accuracy and completeness of gene sets: examples of this approach include the Vega database of vertebrate annotation [2] and some popular model organism databases such as Wormbase [3] and FlyBase [4].

Annotation of other functional genomic features—notably the sequences responsible for regulating gene transcription—has lagged behind. Regulatory elements can be broadly divided into two classes: proximal or core promoter elements that occur close to the initiation site of transcription, and enhancer/silencer elements that act at distance to regulate basal levels of transcription. Both classes of regulatory elements consist of clusters of transcription factor binding sites (TFBSs) [5]. This common architecture suggests that a first step towards regulatory element annotation should be to define a dictionary of motifs that reflects the full repertoire of transcription factor binding specificities. Classically, the binding specificity of transcription factors can be identified using data compiled from DNase I footprinting [6] or in vitro binding site selection experiments [7]; however, data of this kind is only available for a limited subset of transcription factors. For example, just over ten percent of 753 candidate transcription factors in the D. melanogaster genome [8] have annotated binding site data [9]. Moreover, in many cases only one or two sites have been annotated for a given protein, making it hard to build a reasonable model of a factor's binding specificity. Computational methods have existed for more than twenty years that can identify overrepresented motifs in a set of sequences (reviewed in [10]). However, the problem of inferring transcription factor specificity remains intrinsically challenging even for small, well-defined datasets of functionally characterized binding sites [11]. On a genome-wide scale, computational motif discovery methods have typically been applied to 5′ flanking regions of genes grouped by similar expression patterns, with the aim of discovering one or a few factors responsible for controlling coregulated expression. Applying computational motif finders to large sets of unrelated promoter regions from a single genome is a much more challenging task, and previous work in well-studied systems such as *Drosophila* has yielded only a relatively small set of core promoter motifs [12]. If we aim to build a comprehensive motif dictionary for metazoan genomes, it is necessary to scale up the motif discovery process and identify much larger sets of motifs.

Here, we describe the application of NestedMICA [13], a sensitive new computational motif finder, to a large set of D. melanogaster promoter regions. Using this new method, we have so far discovered 120 distinct overrepresented motifs, including good matches to previously reported transcription factor binding motifs (TFBMs) as well as many novel putative motifs. An important feature of our strategy is that the dictionary of motifs is inferred purely from sequence fragments selected from a single genome on the basis of gene annotation (which itself is supported primarily by the alignment of cDNA and EST evidence to the genome sequence). No gene expression or comparative genomic data is used in the selection of motif-discovery input data or in the motif discovery process itself. The latter is particularly important since, in contrast to previous large-scale motif inference efforts [14–17], it means we can assess the quality of the discovered motif set by evaluation against comparative genomic data. Such comparisons offer strong supporting evidence that many of the motifs we have discovered are biologically significant.

### Inferring a Motif Dictionary

The first step in computational motif discovery is to define a good set of search regions. One strategy would be to focus on the most highly conserved non protein-coding portions of the genome, in the expectation that these would be enriched for regulatory elements [18]. However, we chose to avoid this approach, at least for a first round of motif discovery, since the use of comparative genomic data at this point would prevent its use as an independent source of information when we validate our discovered motif set. Instead, we took a more traditional approach of focusing on a set of presumed proximal promoter sequences. To do this, we extracted up to 200 bases of sequence flanking the 5′ ends of annotated genes on D. melanogaster chromosome arm 2L, with some special treatment for very closely spaced genes as described in the Materials and Methods section. Large tracts of low-complexity sequence, such as mononucleotide and dinucleotide repeats, were masked using the dust program (R. Tatusov and D. J. Lipman, unpublished data) with default options. No other preprocessing of the sequences was performed.

In total, this procedure yielded 422 kb of putative promoter sequence from 2,424 genes. Seventy-six percent of genes have annotated UTRs, and we assume that we have obtained true 5′ flanking sequence for most of these. Many D. melanogaster 5′ UTRs are fairly short, with 66% of UTRs less than 200 bases long, so even for the 24% of genes without an annotated UTR, we expect that our set will include at least some 5′ flanking sequence in many cases. Less than 0.7% of promoter regions in this dataset contain a transposable element repeat [19], so it is unlikely that motifs in transposable element sequences contribute strongly to the results presented here. These data represent more than a 2-fold increase in amount of sequence, and a 25% increase in the number of genes analyzed, relative to the primary dataset in [12]. We also note that in contrast to Ohler et al. [12], who investigated motifs on the leading strand from −60 to +40 relative to the transcription start site using MEME [20], we investigated the presence of TFBMs on both strands from −200 to −1.

Our motif discovery strategy was based on the NestedMICA method [13]. NestedMICA is a probabilistic motif finder: it models motifs as position-weight matrices (PWMs) rather than as consensus sequences. PWMs are an established way of modeling the specificity of molecules that interact with nucleic acids [21] and have been shown to be more powerful than simpler representations such as consensus sequences when detecting TFBSs [22].

NestedMICA infers multiple motifs simultaneously. This is distinct from many previous probabilistic motif finders, which have adopted a stepwise approach: finding one motif, masking its occurrences, then finding the next (e.g., [20]). In this regard, NestedMICA shares affinity with methods that perform simultaneous inference of multiple motifs such as the Gibbs Recursive Sampler [23] and CisModule [24]; however, there are no published reports applying these methods to the genome-wide discovery of large numbers of TFBMs in metazoans. Simultaneous motif discovery is likely to maximize sensitivity (see [13]), and it also contributes to the good scalability of the method, since the program does not need to restart the analysis for each additional motif.

NestedMICA is also distinctive in terms of its inference strategy: while previous probabilistic motif finders have used expectation maximization or traditional Monte Carlo methods such as Gibbs Sampling to parameterize their probabilistic models, NestedMICA uses a recent and distinctive Monte Carlo strategy called Nested Sampling (J. Skilling, unpublished manuscripts at http://www.inference.phy.cam.ac.uk/bayesys). This strategy was chosen because we have found it to be effective at finding globally good solutions to the motif-inference problem without requiring heuristics to choose good starting states (see [13]). Another interesting property of Nested Sampling is that it can provide reliable estimates of the evidence term of a Bayesian computation. Bayesian evidence has historically been very hard to calculate, but can be used to perform model comparison (for example, “is this set of sequence data best modeled by a 10-base or an 11-base PWM”) in a manner that correctly penalizes the extra parameters in more complex models [25]. We took advantage of these evidence estimates in the refinement step of our motif discovery pipeline.

While the primary aims in developing NestedMICA were sensitivity and statistical rigor, we also worked hard to maximize performance and scalability. NestedMICA can run on large volumes of sequence data (up to several megabases), and while the run time on large datasets can still be high, this can be made manageable by running the program in a distributed mode that spreads the workload across several machines connected by a fast network. Depending on dataset size and exact configuration, NestedMICA can effectively utilize 10–20 CPUs.

Effective motif-finding strategies require an appropriate background model against which to assess motif overrepresentation. D. melanogaster upstream sequences are known to have compositional biases [26], and it is important that the background model does a good job of capturing these biases. Many motif finders model the background sequences using a single Markov process or, equivalently, a single oligonucleotide frequency table. Where this strategy has been adopted, high-order Markov processes generally give the best results: Thijs et al. recommended a 5th-order (hexanucleotide) model [27]. However, such a model is complex (3,072 free parameters for a 5th-order Markov chain) and potentially hard to train: biologically meaningful regulatory motifs could be captured by such a high-order background model, preventing their detection in a subsequent motif inference step, and therefore it would be necessary to select truly nonfunctional sequences for background model training. An alternative approach is to relax the assumption that the sequence is generated by a single Markov chain. NestedMICA implements a family of background models where each base of the sequence is generated by one of several possible Markov chains. We call these mosaic models, since they treat large sequences as mosaics of compositionally distinct regions. We have previously shown that a mosaic of four order chains can better model mammalian promoter sequences than a single higher-order chain, while requiring fewer free parameters [13]. We used a similar strategy here and randomly split the set of 5′ flanking sequences on chromosome arm 2L in half to give independent “test” and “training” sets, then used the training portion to optimize a range of background models—with between two and eight classes—using the makemosaicbg program from the NestedMICA package. The results were very similar to those shown in [13], except that on these D. melanogaster sequences the optimal model consisted of six order classes. We selected this six-class model as the basis for our large-scale motif inference. As in the mammalian case, we saw classes modeling neutral, purine-rich, and pyrimidine-rich regions. We also saw *Drosophila*-specific classes for A/T-rich, C/A-rich, and G/T-rich regions. There was no equivalent of the mammalian G/C-rich sequence class, which associated primarily with CpG islands.

For the analysis presented here, we inferred 120 motifs (Figure S1, statistics also included in Table S1) from the chromosome arm 2L 5′ flanking sequences. The NestedMICA method currently requires that the motif length be specified a priori. For this initial production run, we requested 12 base motifs: long enough to represent the core length of most known *Drosophila* TFBMs. Motif inference, following the procedure under Materials and Methods, took approximately four weeks on eight Pentium IV processors (2.8 GHz clock speed). We have derived several other sets of motifs, from the same set of chromosome arm 2L sequences with slightly different NestedMICA parameters as well as also from other D. melanogaster chromosome arms. Using the motif comparison strategy described in the Materials and Methods section, we always see a large overlap between independently trained sets of motifs (unpublished data). Discovery and refinement of the motif dictionary is an ongoing process, and in the future we plan to scale up the procedures described here and explore strategies for merging overlapping motif sets to produce a single comprehensive set of TFBMs.

All the initially discovered PWMs were 12 bases long, but in many cases several positions towards the edge of the motif had very low information content, suggesting that the PWM was a model of an underlying motif less than 12 bases long. Therefore, we individually retrained each motif, following the refinement and trimming procedure under Materials and Methods. In 54 out of 120 cases, this led to a shorter final PWM. Finally, in six cases where we were confident that the discovered PWM correspondended to a previously reported, named motif (as discussed at length below), we chose to reverse-complement the inferred motif PWM to correspond with the previously reported orientation. None of our subsequent analyses are sensitive to motif orientation, so this manipulation should have no effect except in terms of motif display.

The motif dictionary contained many motifs with specificity towards A/T rich sequence: 54 out of 120 motifs preferred to match sequences that are more than 66% A/T. This was not due to inability of our motif inference strategy to find G/C-rich motifs: there are several motifs in the set with very strong preferences towards G/C rich sequences. It is possible that there might have been some bias in our inference procedure that leads to preferential detection of A/T rich motifs, but we doubt this explanation: noncoding D. melanogaster sequence as a whole is A/T rich (61.6% A/T on average across our set of 5′ flanking regions), so it would not be too surprising to find that many of the most common regulatory elements might also be A/T rich. Also, our background model was trained on the same set of 5′ flanking sequences and contains a class that modeled regions of sequence with a high A/T content. Thus, the overall high A/T content in 5′ flanking regions was accounted for during the motif inference process, which searched for overrepresented motifs relative to this background model.

We attempted to define an optimal score cutoff to use when scanning bulk sequence with each of our inferred PWMs. To do this, we scanned the training set of 5′ flanking sequences as described under Materials and Methods, and subdivided the matches by score. For each 1-bit score interval (bin), we assessed overrepresentation of motif matches relative to what might be expected given our mosaic background model (see Materials and Methods). We saw significant (*p* ≤ 0.05 in a binomial test) overrepresentation in high-scoring bins for 118/120 motifs, and were thus able to select a score cutoff. One limitation of this method is that its resolution is limited to the width of the bins used to subdivide the matches—in this case 1 bit. Narrower bins might help, but would reduce the power of the statistical tests used to assess overrepresentation. Applied to our motif set, this method suggested cutoffs between −1 and −8 bits relative to the maximum possible score for each motif. This wide variability implies that using a common threshold for all motifs would not be optimal, and also suggests that some DNA-binding factors might be more tolerant of variation in their binding sites than others. We use these suggested cutoffs in several of our subsequent analyses. It is worth noting that this procedure also indicates that all but two of our inferred PWMs are significantly overrepresented relative to the background model. This should not be surprising—overrepresentation was, after all, the criterion for motif inference—but it does give us additional confidence in the inferred motif set. It also suggests that the two motifs that weren't significantly overrepresented (TIFDMEM0000001 and TIFDMEM0000043) should be treated with some caution.

All our discovered PWMs are also available in release 1.2 of Tiffin, a database of sequence motifs. Tiffin can be browsed via a web interface, which also permits export of the motif PWMs in a variety of machine-readable formats: http://servlet.sanger.ac.uk/tiffin.

Tiffin assigns IDs to computationally discovered motifs in the same spirit as the IDs assigned to Ensembl gene predictions. As with Ensembl, the intention is to maintain IDs wherever possible as the motif collection grows and improves. We use Tiffin IDs throughout this paper when referring to specific motifs in our discovered set. A typical Tiffin ID is TIFDMEM0000040. This is made up of a database identifier (TIF for Tiffin), a three-letter species code (DME for D. melanogaster), a one-letter object type (M for motif), and finally a numerical identifier. This syntax closely matches that used by Ensembl, and where possible Tiffin will use the same species codes.

### Assessment of the Motif Dictionary

Given that little is currently known about the binding specificities of most transcription factors, it is difficult to evaluate whether discovered motifs are indeed authentic. Here, we consider four distinct lines of evidence: comparison with previously reported sequence motifs, analysis of cross-species conservation, analysis of motif position in the genome, and association between motifs and gene expression pattern. All four of these analyses offer support for a substantial fraction of our discovered motifs, suggesting that our motif-discovery approach can successfully recover motifs that are predictive of functional sequences. Alongside the comparison step, we also address issues of possible redundancy within the motif collection.

### Comparison with Known Motifs

**Figure 1:**
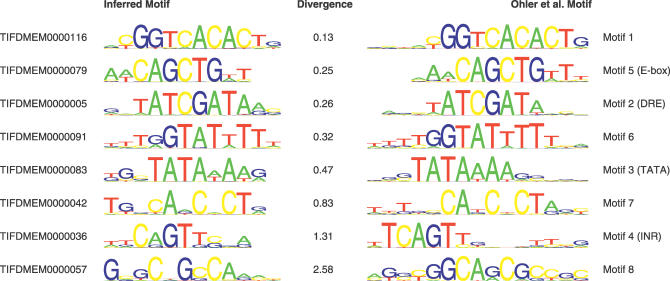
Best Reciprocal Matches between the NestedMICA Promoter Motif Set (Left) and the Ten Core Promoter Motifs Reported by Ohler et al. (2002) (Right) Scores in the central column represent divergence scores (see Materials and Methods). All matches were found to be highly significant (*p* < 0.001 using the shuffling test described under Materials and Methods).

The pioneering study by Ohler et al. (2002) demonstrated the possibility of large-scale probabilistic promoter motif inference in metazoans, and generated a list of top ten motif PWMs that are overrepresented in D. melanogaster core promoter regions [12]. We assessed whether this limited set of highly abundant motifs could be recovered by NestedMICA while simultaneously searching for a much larger set of motifs. To do this, we measured the divergence between each of the ten reported promoter motifs and each of the 120 motifs discovered by our strategy, using the divergence function described in the Materials and Methods section. We then searched the resulting divergence matrix for best reciprocal hits: pairs of motifs where each is the others' best match. This is closely analogous to the strategy used to define orthologous genes between two genomes. Finally, we assessed the statistical significance of each match by repeating the comparison using shuffled PWMs: the fraction of cases where a shuffled PWM can give an equal or better score gives an empirical *p*-value for the comparison. We found reciprocal best matches for eight out of ten of the Ohler et al. (2002) motifs (Figure 1), including well-established promoter sequences such as the TATA, DRE, and INR motifs. All eight of these matches were highly significant (*p* ≤ 0.001) In seven out of ten cases, visual inspection leaves little doubt that the motifs are essentially identical, while the final and most divergent case (TIFDMEM0000057 versus Motif 8) shows some differences. Both of the previously discovered motifs with no best reciprocal match in our set (DPE and MTE) have been shown to be located primarily downstream of the transcription start site [12], so it is not surprising that we did not find them in our set. We note that an independent set of 15 consensus motifs derived from positionally biased octomers in *Drosophila* promoters recently reported by FitzGerald et al. (2006) [26] overlaps substantially with the Ohler et al. (2002) set and therefore also with the subset of NestedMICA motifs in Figure 1.

For a subset of developmentally regulated transcription factors in *Drosophila,* a reasonable amount of experimental evidence from SELEX-like methods [7] and DNase I footprinting assays [6] is available to infer their binding specificities. From such empirical data, it is possible to derive PWMs that should be a good reflection of the binding specificity of the protein in question, at least in vitro. However, it is important to note that the motifs learned from in vitro binding of purified protein to naked DNA may not accurately represent those obtained from in vivo conditions. We expect motifs inferred directly from genomic sequences to differ slightly from in vitro sequences, and in some cases may better reflect the in vivo binding specificity of a transcription factor.

For this analysis, we used two reference collections of experimentally supported PWMs. The first is a set of PWMs we have learned from the FlyReg database of DNase I footprints [9]. For each factor in the FlyReg dataset with at least five footprints, we attempted to infer a single optimal PWM using NestedMICA, as described in the Materials and Methods section. From an original set of 52 factors, we obtained a set of 30 optimal PWMs for known *Drosophila* transcription factors (Figure S2) that we can compare to the set of 120 motifs learned from bulk genomic DNA. The second dataset used for evaluation is the JASPAR CORE collection of PWMs [28], which are derived primarily from SELEX experiments or other compiled experimental data. This set includes 123 motifs (database accessed 05/07/2006) from a variety of species, including some from *Drosophila,* but also many vertebrate, yeast, and plant motifs. Many additional *Drosophila* motifs exist in the literature that are not present in JASPAR, and thus we extended the JASPAR CORE set of motifs to include an additional 49 SELEX and consensus motifs for *Drosophila* transcription factors curated from primary publications (http://bioinf.man.ac.uk/bergman/data/motifs). This resulted in a set of 172 motifs of which 62 are derived from *Drosophila* transcription factors (Figure S3).

**Figure 2:**
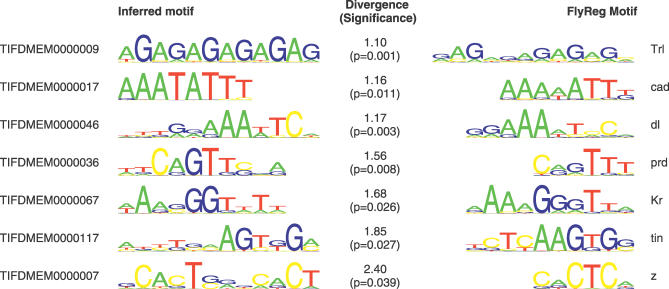
Statistically Significant Best Reciprocal Matches between 120 Discovered Motifs and Motifs Inferred from *Drosophila* DNase I Footprint Data

**Figure 3:**
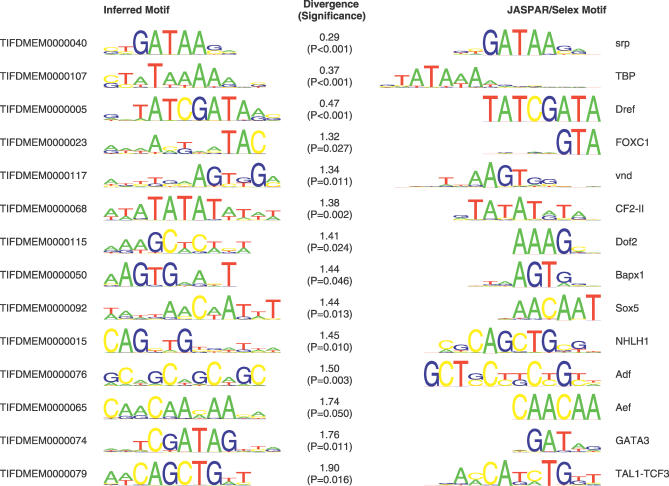
Statistically Significant Best Reciprocal Matches between 120 Discovered Motifs and Motifs from Extended JASPAR CORE

We performed a reciprocal-best-hits assignment between 120 discovered motifs and both of these known TFBM sets, using the same divergence measure and significance-testing procedure as before. We applied a significance threshold of *p* ≤ 0.05 for all comparisons. As shown in Figures 2 and 3, we see a number of very good matches in both sets of experimentally derived PWMs. One striking match shown in Figure 2 is between TIFDMEM0000009 and *Trithorax-like (Trl),* the gene encoding the GAGA factor, a protein involved in activating gene expression by influencing chromatin structure (reviewed in [29]) and which is known to bind a large number of genomic regions [30]. A second striking match shown in Figure 3 is between TIFDMEM0000040 and *serpent (srp),* a GATA factor necessary for the development of the amnioserosa, fat body, endoderm, and blood cells (reviewed in [31]). Known binding sites for both *Trl* and *srp* are found within 200 bp of the TSS of their respective genes [9], and therefore binding sites for these developmentally regulated transcription factors might be expected to be enriched in our dataset. In total, we saw seven matches to FlyReg, 14 matches to the extended JASPAR CORE, and eight matches to the motifs from Ohler et al. (2002) [12]. Accounting for redundancy between these sets, we obtained a set of 25 inferred motifs which significantly match a known TFBM.

We further investigated the similarities between discovered and previously known motifs by considering the fraction of common predictions between two similar PWMs. Given sets of base positions covered by predictions from two motifs, *B*_1_ and *B*_2_ *,* we define the overlap between them as:

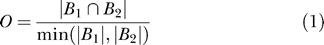
i.e., *O* = 0 when the two motifs match completely distinct sets of positions, while *O* = 1 if one motif is matching a subset of the other's predictions. This latter property should mitigate the effect of any potential errors made when setting score cutoffs for each of the motifs. We used this measure to compare each of the 25 known motifs in our set with the closest existing PWM from the three sets above. Inferred PWMs were scanned across the whole sequence of D. melanogaster chromosome arm 2L using the optimal score cutoffs we defined previously. We set score cutoffs for the corresponding existing PWMs using the same strategy, then calculated prediction overlaps for 22 pairs of PWMs (for three of the existing PWMs, we were unable to define an optimal cutoff). In 12 out of 22 cases, the predictions matched rather closely (*O* ≥ 0.5, and in three cases *O* ≥ 0.9). In eight out of 22 cases, 0.1 <*O* ≤ 0.5. This still represents a substantial overlap, but the majority of predicted bases are different. This suggests that the inferred PWM in these cases might not be doing a good job of modeling the same binding specificity as the existing known motif, which could represent a failure of the motif inference process. Alternatively, these cases might also suggest the existence of a family of similar, but not identical, motifs—perhaps targeted by a family of related DNA-binding proteins. Finally, two out of 22 pairs show little or no overlap between sets of predicted sites.

These results confirm that NestedMICA can simultaneously recover many good PWMs from large genomic datasets, for both core promoter motifs and motifs for developmentally regulated transcription factors with characterized binding specificities. Nevertheless, 95 of the motifs we have discovered were not assigned to a known TFBM by this analysis. This result may not be surprising: characterization of a transcription factor's binding specificity is a complex and laborious process, and only a relatively small subset of known factors have been fully studied. Therefore, it seems reasonable to propose that many of the remaining discovered motifs will be good models for the binding specificities of as-yet-uncharacterized transcription factors.

### Assessment of Redundancy in the Motif Dictionary

**Figure 4:**
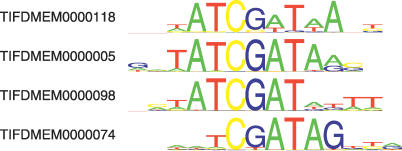
The Largest of 13 Clusters of Similar Motifs Clusters were identified by finding significant matches in an all-against-all comparison of 120 inferred motifs.

Our set of 120 inferred motifs contains several PWMs that are visually quite similar (for examples, see Figure 4). To address the question of whether our motif set includes possibly redundant motifs, we applied a similar comparison strategy to that described above to perform an all-against-all comparison of the 120 PWMs. Using a significance threshold of *p* ≤ 0.05, we found 25 significant similarities between 31 of the 120 motifs. These 31 motifs formed 13 clusters, suggesting that 18 out of 120 motifs might be redundant. The largest of these clusters had four members, which are shown in Figure 4. We also analyzed the overlaps between predicted sites for our motifs, using the same strategy as above. As in the comparison of known motifs, significantly similar PWMs do not always predict strongly overlapping sets of sites: indeed, only one of the 25 pairs had an overlap score of *O* > 0.5. Therefore, it seems possible that some of these similar motifs are not truly redundant but might instead represent binding specificities for related—but not identical—transcription factors.

The question of motif redundancy is important when classifying our inferred motifs as known or novel. While 25 out of 120 PWMs are significant best-reciprocal-matches to known motifs from one of the existing motif sets described above (and are therefore classified as “known”), an additional eight related PWMs show significant similarity to one of these known motifs. This leaves 87 out of 120 PWMs (80 out of 102 if we assume that all similar motifs are in fact duplicates) that we classify as novel.

### Conservation of Discovered Motifs

Functional binding sites are likely to be subject to purifying selection and thus should exhibit a reduced rate of sequence evolution. This is based both on the observation of increased levels of conservation in known TFBSs relative to their background sequences [32,33] and the intuition that losing elements responsible for gene regulation may often be deleterious [34]. Of course this does not mean that all regulatory elements are under strict purifying selection, and indeed there are good examples of divergence in regulatory element function [35], as well as conservation of regulatory function with underlying binding site turnover at the sequence level [36]. Nevertheless, increased conservation of predicted TFBSs provides evidence for functional constraint [33].

**Figure 5:**
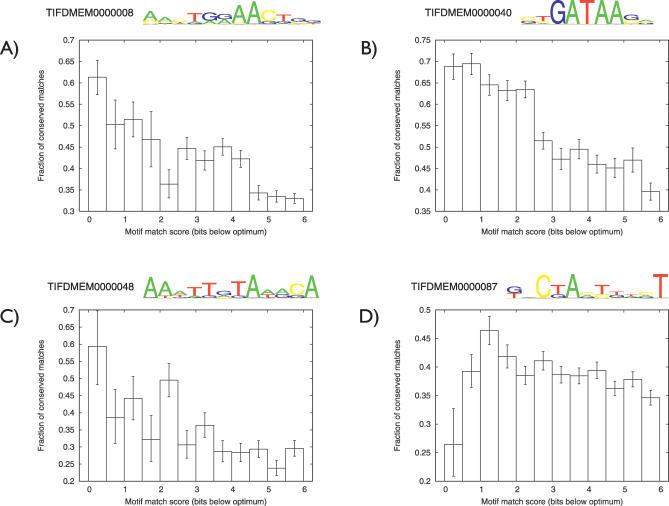
Example Plots Showing the Fraction of Motifs in Each Score Bin That Are Conserved among Three *Drosophila* Species Increasing bin numbers indicate worse scores. The error bars in this plot indicate 99% confidence intervals calculated from a β distribution. (A–C) Show results for motifs that are significantly overconserved. (D) Shows an underconserved motif.

To test whether motifs in our set show signatures of evolutionary constraint among *Drosophila* species, we studied patterns of motif conservation in a large set of orthologous non protein-coding alignments. Alignments were available genome-wide, but to avoid any possible overfitting artifacts, we discarded the subset of alignments matching D. melanogaster chromosome arm 2L. Since we are more confident of the non protein-coding sequence alignment between closely related species [37], we concentrated on testing conservation between D. melanogaster and two closely related species, D. simulans and D. yakuba. For each match to the D. melanogaster genome, we looked for matches of the same motif to orthologous positions in all three genomes. We then stratified all the D. melanogaster matches of a given motif by decreasing bit-score. In each bin, we calculated the fraction of sites where a prediction was present in all three species (score ≥7 bits for all motifs. This cutoff is less stringent or equal to the optimal cutoffs chosen for all but one of our inferred motifs). In many cases, we saw striking correlations between motif score and degree of conservation, as shown in Figure 5. While the most common pattern is for high-scoring motifs matches to be more conserved, in a few cases we saw a strong inverse correlation (e.g., TIFDMEM0000087), with the strongest matches being substantially less conserved. These underconserved motifs are intriguing, since such a distribution of conservation seems improbable if the motif wasn't associated with some function.

In total, 78 out of 120 motifs showed statistically significant (*p* ≤ 0.001 in a test) excess conservation for the highest-scoring matches compared with the lowest-scoring matches. At this significance level, we expect a false discovery rate of less than one motif to show an excess of conservation for high-scoring matches, and thus we interpret this result as strong evidence for the majority of discovered motifs being preferentially maintained by purifying selection. A further 22 motifs showed significant underconservation, and the remaining 20 had little or no correlation between score and conservation. This does not conclusively show that they do not reflect functional binding sites, but does suggest that perhaps a relatively small fraction of the total matches to the genome are functional for these motifs. Taking a conservative interpretation of these results (i.e., that only motifs positively correlated with increased sequence conservation have any power to recognize functional sites), this suggests that 65% of our discovered motifs may reflect functional recognition sequences.

### Positional Biases of Discovered Motifs

It is widely expected that motifs that are functional constituents of promoters should be specifically overrepresented in promoter regions (i.e., close to, and especially upstream of, the transcription start sites) relative to the rest of the genome. This was our main justification for using 5′ flanking sequences when inferring the motif dictionary. Positional bias in the distribution of motifs relative to TSSs has been used in the past in *Drosophila* for characterizing discovered motifs [12] and as an objective function for motif discovery [26].

**Figure 6:**
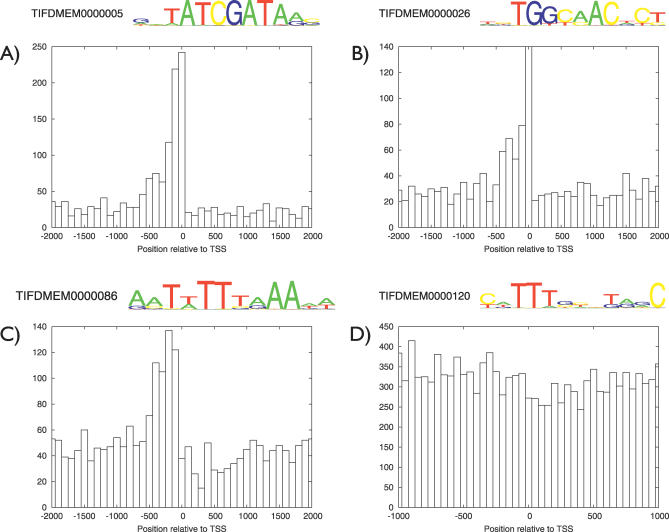
Position Distributions of Matches to Selected Motifs around Transcription Start Sites on Chromosome Arm 2R (A,B) Show strong upstream peaks with different shapes. (C) Shows a broader (but still highly significant) upstream peak and some underrepresentation immediately downstream of the TSS. (D) Shows results for a motif with little or no positional bias.

Since we used only one chromosome arm for the initial motif discovery process, we had access to many independent promoter sequences for testing purposes. To investigate positional biases of our predicted TFBMs, we scanned the whole sequence of D. melanogaster chromosome arm 2R, using the score cutoffs defined on the basis of overrepresentation tests. For each motif, we recorded the number of matches in 100-base windows relative to the starts of all annotated transcripts. Many motifs showed nonuniform distributions relative to transcription start sites, but the exact distribution varied, with some motifs located very close to the TSS while others showed much broader peaks (c.f., [26]). In many cases, there was also a trough just downstream of the TSS, but again the magnitude and width varied. Several representative examples are shown in Figure 6.

To evaluate the significance of positional biases, we counted motif matches overlapping 400 (200 upstream, 200 downstream) 100-base windows relative to the transcription start sites. In 70 out of 120 cases, the highest peak occurred in the 400 bases immediately upstream of the TSS. We consider these motifs to have significantly nonuniform position distributions (*p* ≤ 0.01). We would expect a false discovery rate of slightly more than one of our 120 motifs to show a positional bias in this analysis.

There are known compositional biases in D. melanogaster promoters [38]: in particular, an overrepresentation of A/T both upstream and (to a slightly lesser extent) downstream of the TSS. It is possible that this mononucleotide frequency bias might explain at least some of the observed motif position bias, so positional bias is not in itself compelling evidence that a motif does in fact represent the binding specificity of a transcription factor. However, not all positionally biased motifs are A/T-rich (e.g., TIFDMEM0000026, see Figure 6B). It is also possible that causality runs in the opposite direction: promoters could be A/T-rich primarily because they are enriched with large numbers of A/T-rich motifs. In any case, we believe that a strong association between any motif and transcription start sites suggests that a motif is biologically interesting, even if it does not confirm its status as a TFBM.

### Association of Discovered Motifs with Gene Expression Patterns

We would like to discover the biological function for each of our motifs. Functional annotation of motifs offers an extra line of evidence that they are biologically relevant, and may also prove useful in understanding the contribution that individual motifs make to regulatory elements in the genome. Previous work has attempted to associate discovered motifs with Gene Ontology terms or with microarray expression data for genes containing motif instances [12,26]. Here we base our functional annotation on a dataset of whole mount in situ hybridization experiments, which includes annotated expression patterns of about 3,000 genes in developing *Drosophila* embryos [39]. Although the primary content of this database is a set of images showing where each target gene is expressed, this image atlas is accompanied by a curated set of labels that use terms from a controlled vocabulary (ImaGO) to describe where each gene is expressed at each developmental stage where it was seen.

Genome-wide scanning of PWMs is widely considered to give many false positive matches [40]. For this analysis, it poses an additional problem: given a PWM match to bulk genomic sequence—especially in intergenic regions—it is hard to predict which of the nearby genes is the most likely regulatory target. We therefore focused once again on probable promoter regions: 200 bases upstream of each gene. We scanned 200 bases of 5′ flanking sequence for all annotated D. melanogaster genes with all 120 motifs, using the score thresholds defined previously, then counted the number of times each motif was associated with each term in the ImaGO vocabulary, either directly or via the hierarchy of terms in the ImaGO ontology. For this analysis, we counted all co-occurrences, including cases where multiple matches to a given motif occur upstream of a given gene. We tested the significance of each positive association by removing the motif ID labels from all the motif match records, shuffling all the labels, then reattaching them randomly to match records. By repeating this shuffling process many times (in this case, 5,000,000 iterations) and recording the occasions when a given motif-to-expression association is equally or more abundant in the shuffled data than in the real data, we can obtain empirical *p*-values that describe how often each observed association would have occurred by chance.

**Figure 7:**
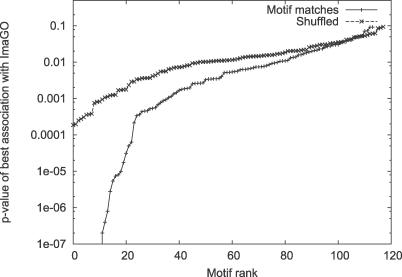
Significance of Correlation between Motifs and ImaGO Terms, in Rank Order The “shuffled” trace gives an indication of the likely false discovery rate at a given *p*-value significance threshold.

The *p*-values calculated by this method are not directly useful, since we have performed repeated testing of each motif against each term in ImaGO. One way to correct for this would be to apply a Bonferroni correction by multiplying each *p*-value by the number of terms in ImaGO. However, such a correction would give an overly conservative picture, since many ImaGO terms are highly correlated with one another, so in practice not every test is independent. Instead, we performed another run of the association process using shuffled motif IDs rather than the real labeling giving an empirical view of the false discovery rate we would expect from this method if motifs matched randomly around the genome. For each of these two runs—real and shuffled motif labeling data—we found the lowest *p*-value for each motif, out of all the ImaGO terms. These are plotted in rank order in Figure 7.

**Figure 8:**
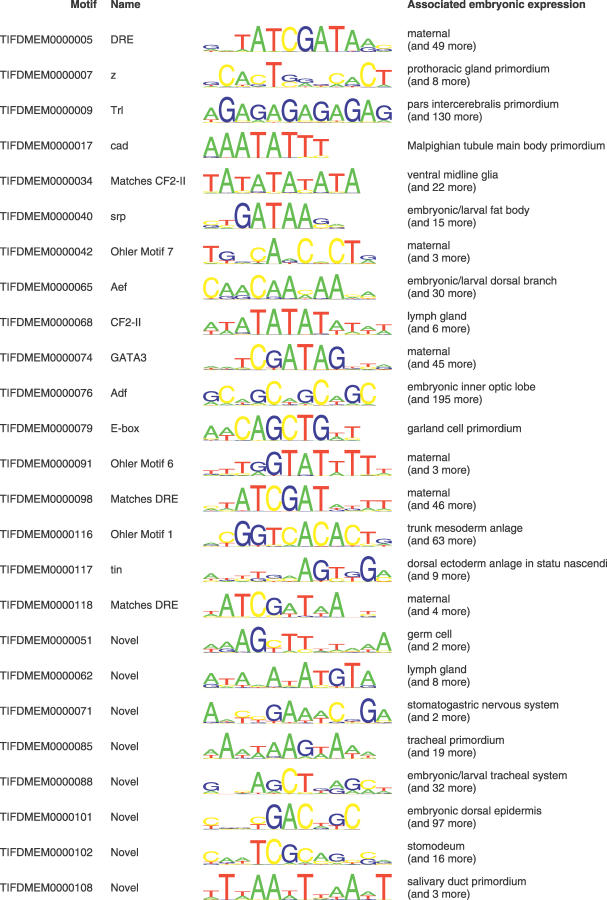
Motifs with Significant Correlations to Embryonic Expression Patterns A top-scoring ImaGO term is shown for each motif.

From this analysis, we see that many motifs associate strongly with at least one ImaGO term. We take this as strong evidence that a large fraction of the discovered motifs are involved in time- or tissue-specific transcriptional regulation during the embryonic stages 1 through 16. Moreover, a subset of these have associations that are very much stronger than any associations seen in the randomized set. Setting a threshold of 3.5 × 10^−4^—at which we would expect approximately one false discovery—25 motifs have at least one association that we consider significant, including eight novel motifs. These 25 motifs are shown in Figure 8. The largest sets of associations are observed for the TIFDMEM0000009/*Trl* motif (*n* = 131) and *Adf*-like motif TIFDMEM0000076 (*n* = 196), suggesting potentially widespread roles in embryonic development for the GAGA factor and a putative factor that may bind TIFDMEM0000076. We found that the TATA and INR core promoter motifs discovered here do not have significant associations with ImaGO terms, which is consistent with previous results that these motifs are used preferentially by genes that are active in the adult [26]. The full set of ImaGO terms for each of these 25 motifs can be browsed interactively at http://servlet.sanger.ac.uk/tiffin.

A number of interesting associations emerge from this analysis. Foremost is a set of associations between TIFDMEM0000040 and nine ImaGO terms that fall into two categories relating to the development of the fat body (fat body specific anlage, fat body/gonad primordium, embryonic/larval fat body, fat body, embryonic/larval adipose system, and adipose system) and development of the amnioserosa (extraembryonic structure, amnioserosa anlage *in statu nascendi,* amnioserosa). Since the fat body is mesodermal in origin and the amnioserosa is an extra-embryonic tissue, we interpret this result as two independent biological associations of the TIFDMEM0000040 motif with two independent sets of related ImaGO terms. As noted above, TIFDMEM0000040 is an almost perfect match to the PWM for the gene *srp,* which is required for the ongoing differentiation and maintenance of the fat body [41] and amnioserosa [42]. If the TIFDMEM0000040 motif reflects *srp* specificity, the association of TIFDMEM0000040 with many genes is consistent with the hypotheses that *srp* activates a “large battery of early and late fat-body genes” [41] and may function as a “selector gene” [31].

## Results/Discussion

Finding regulatory elements in large genomes remains one of the hardest, and also one of the most exciting, problems in contemporary genome biology. Here we have shown that simultaneous probabilistic motif inference using NestedMICA can successfully be applied to large sets of unrelated promoter sequences in metazoan genomes. We have produced a dictionary of 120 motifs in an attempt to capture a significant fraction of the common promoter motifs in *Drosophila*. While our set still falls well short of the estimated 753 transcription factors in the D. melanogaster genome [8], we expect that scalability improvements in the NestedMICA algorithm, and the use of larger datasets—should close that gap over time.

We offer several lines of independent evidence that support the validity of many of the 120 motifs discovered here. First, we find significant matches to eight out of ten core promoter motifs found previously in a smaller dictionary of computationally derived motifs [12]. The two unmatched motifs were not expected to be recovered in our analysis of upstream flanking regions, since they have been shown to be preferentially located downstream of the TSS [12]. Thus, we can recover all these previously discovered upstream promoter motifs while simultaneously inferring a much larger dictionary. Some of these additional motifs in our dictionary are quantitatively and qualitatively very similar to experimentally derived binding transcription factors motifs (Figures 2 and 3), including matches to developmentally regulated transcription factors, such as *srp*. Together, these results demonstrate that running NestedMICA on large sets of sequences is an effective way of simultaneously recovering valid binding motifs for both basal and developmentally regulated transcription factors.

**Figure 9:**
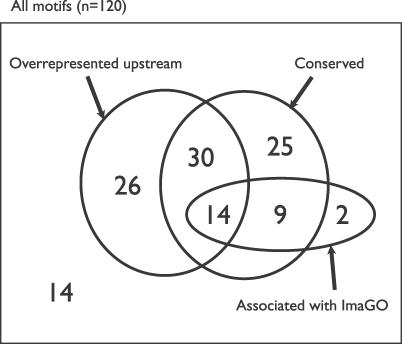
Summary of Lines of Evidence Supporting 120 Motifs Discovered in This Study

In addition to recovering known motifs, 87 of the motifs discovered in this study have no significant match—directly or indirectly—to any of the three reference motif sets used here, suggesting that NestedMICA can also discover novel motifs. We believe that a large proportion of these novel motifs are predictive models of functional sequence elements, as the majority of the motifs discovered here are either preferentially conserved (78/120, 65%, including 22/25 known and 49/87 novel motifs) or preferentially located upstream of transcription start sites (70/120, 58%, including 14/25 known and 50/87 novel motifs). In total, 106 out of 120 motifs (88%) are supported by at least one of these two analyses. Furthermore, many motifs are preferentially located near genes with similar expression patterns in D. melanogaster embryos, and for 25 of these (14 of which were previously known and eight of which we believe are novel) we can make statistically significant associations to one or more ImaGO controlled vocabulary terms used to annotate the in situ gene expression atlas. A summary of the sources of evidence supporting each of our predicted motifs is shown in Figure 9.

Integrating results from our study with previous analyses of promoter motifs in D. melanogaster [12,26] reveals that diverse computational strategies yield nearly identical sets of core promoter motifs, including classical promoter motifs such as the TATA box and INR. In addition, all three studies identify the DRE and E-box motifs, suggesting that proteins that bind these motifs play an important role in regulating a large number of *Drosophila* genes. All three studies also consistently recover three uncharacterized motifs (TIFDMEM0000116/Motif 1/Dmv4, TIFDMEM0000091/Motif 6/Dmv5, TIFDMEM0000042/Motif 7/Dmv3) that are likely to be widely used binding sites for as-yet unknown transcription factors. Intriguingly, we find strong associations for all three of these unknown motifs (and for DRE) with ImaGO terms, including maternal expression. These associations support previous results indicating that the presence of these motifs positively correlate with female germline expression [26]. Moreover, we find one of these unknown motifs (TIFDMEM0000116/Motif 1/Dmv4) is strongly associated with multiple ImaGO terms, implicating a role in mesoderm and muscle development. Discovering the factors that bind these putative TFBMs may uncover new core promoter selectivity factors in *Drosophila*.

The strong association of TIFDMEM0000040 with genes expressed in the fat body and amnioserosa demonstrates that we may be able to discover and annotate the function of developmentally regulated TFBMs in upstream flanking regions using NestedMICA in conjunction with the ImaGO controlled vocabulary. However, it is difficult to unambiguously interpret these associations as deriving solely from the *srp* gene, even though the TIFDMEM0000040 shows a best hit to the *srp* PWM, and *srp* is known to be involved in the development of both the fat body and amnioserosa [41,42]. The *Drosophila* genome contains five recognized GATA factors, which are likely to share similar binding specificities, as has been shown directly for two genes, *srp* [43] and *pannier (pnr)* [44]. In addition, *pnr* has been shown to be expressed in the amnioserosa [45], and cells of the amnioserosa die in *pnr* mutant embryos [46]. Thus, the TIFDMEM0000040–ImaGO association may in fact derive from a composite signal of both *srp* (in the fat body and amnioserosa) and/or *pnr* (in the amnioserosa). Likewise, all five GATA family members may contribute to the signal of TIFDMEM0000040 overrepresentation in promoter regions. This example highlights a general problem in any large-scale motif inference effort—resolving the many-to-one mapping of factors with related specificities to individual motifs—a problem that should be less severe in model organisms such as *Drosophila* that have fewer paralogues per transcription factor gene family [47]. The discovery in our motif dictionary of a number of apparently similar PWMs that nevertheless match to substantially distinct sets of genomic sites suggests that computational methods may be able to distinguish between the exact binding specificities of related transcription factors, but this remains a topic for future research.

We acknowledge that our strategy of discovering motifs purely from 200 bp of 5′ flanking regions is a significant limitation: in particular we recognize that binding sites for proteins that interact exclusively with distal enhancer/silencer elements rather than proximal/core promoter regions are likely to be overlooked in this analysis. Underscoring this point is the fact that the majority of known motifs for developmentally regulated factors are not recovered here, suggesting that these factors do not bind preferentially to the 200 bp upstream of their target genes. Given the computational challenge presented by a whole-genome motif discovery experiment, we believe that the approach taken here is a simple and robust intermediate strategy for obtaining sequences enriched in a variety of TFBMs, and our resulting motif dictionary—including 87 novel motifs, eight of which have significant associations to embryonic expression patterns—appears broadly to support this decision.

Looking to the future, the most significant question remains that of how best to use a motif dictionary to scan the genome and to annotate functional binding sites. Simply scanning bulk DNA with PWMs tends to yield many false positive matches, even when using relatively stringent score thresholds. Searching for clusters of predicted binding sites has been shown to improve regulatory element detection [48], but does not itself solve the problem of annotating individual binding sites. It is well-known that comparative data generally can improve functional genomic predictions, and sequence conservation specifically has been shown to enhance TFBS annotation [49]. At the time of writing, genome sequences are available for 12 *Drosophila* species, so they should offer a good platform to investigate comparative approaches to TFBS annotation. Improving the specificity of TFBS annotation should reduce the false discovery rate when performing analyses such as the comparison with ImaGO terms presented here, and indeed such analyses may represent a good initial in silico test for new TFBS annotation methods.

## Materials and Methods

### Genome sequence and annotation.

We used versions 3 and 4 of the Drosophila melanogaster genome sequence from BDGP [50] and corresponding curated gene annotation from FlyBase [4]. Sequence and annotation was extracted from the Ensembl database [1].

Genome sequence from other drosopholids was obtained via the Drosophila Assembly/Alignment/Annotation portal http://rana.lbl.gov/drosophila/

### Multiple sequence alignments.

Multiple sequence alignments between the noncoding genomic sequences of *Drosophila* species were obtained from http://rana.lbl.gov/drosophila/alignments_eisenlab.html.

Briefly, these alignments were produced by using synteny information plus the results of BLAST [51] comparisons of exon sequences to define orthologous exons, then using MLAGAN [52] to align the regions between each adjacent pair of exons from all available genomes.

### Extraction of 5′ flanking sequences.

We considered all gene starts on chromosome arm 2L in version 3 of the D. melanogaster genome annotation. Where multiple transcripts were annotated for a single gene, the start of the most upstream transcript was considered to be the gene start. For each gene start site, we attempted to extract 200 bases of 5′ flanking sequence. However, if this region overlapped another gene, it was truncated such that no sequence was extracted from within annotated genes. If this criterion meant that the length of the 5′ flanking region fell below 101 bases, it was discarded. In some cases, two gene start sites in opposite orientations fell close to one another (“divergent genes”). When this meant that two regions would overlap, they were merged into a single region such that no sequence was duplicated in the final set.

### Large-scale motif inference.

Large sets of motifs were inferred from 5′ flanking sequences using the motiffinder program from version 0.7.0 of the NestedMICA package. The following options were used: (1) -numMotifs 120: desired number of motifs; (2) -targetLength 12: desired PWM length, (3) -expectedUsageFraction 0.1: specifies a prior belief that many motifs will be relatively rare in the input sequence set; (4) -revComp: allow motifs to occur in either orientation; (5) -mixtureUpdate weakResample: enables an optimization for faster estimation of NestedMICA's internal matrix that indicates which motifs are present in which input sequences

In addition, NestedMICA has various options that control communication between nodes on a network, and the creation of checkpoint files that periodically store the state of the ongoing computation. None of these options directly affect the final outcome, but they are important for fast and reliable completion of long-running processes. We refer interested readers to the NestedMICA documentation (see Availability section).

### Searching and scoring motif matches.

A motif PWM is a generative model for a small fragment of sequence; therefore, the natural score for a PWM *W* at position *p* in sequence *S* is:

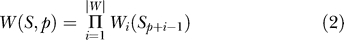
For convenience, we perform two transformations on this basic scoring function. First, in common with most workers in the field, we use (bit) scores. Second, to compensate for the wildly different magnitudes of score from different motifs, we subtract the highest possible bit score (i.e., the score that would be received by a sequence fragment that consisted of the most likely symbol at each position), so that the highest possible renormalized score for each motif is always 0.

### Motif refinement and trimming.

Each motif in the initial discovered set was scanned across the 5′ flanking sequences from chromosome arm 2L. At each position where the motif matched with a score ≥−6, we extracted a short sequence containing the motif match (always 12 bases in this case) plus ten bases of flanking sequence on either side.

NestedMICA requires that the target motif length be specified when the program is run, and does not attempt to optimize PWM length internally. NestedMICA can, however, output the Bayesian evidence for training a particular model configuration (including PWM length) on a given set of sequences, estimated using the Nested Sampling method. We therefore performed multiple runs of NestedMICA with target motif lengths varying from four to 12 (otherwise using default options) and selected the model with the highest evidence. For this analysis, we used the same background model as originally used for large-scale motif discovery.

### Picking score cutoffs for PWMs.

PWMs were scanned across the training set of chromosome arm 2L 5′ flanking sequences as described above, using an extremely lenient threshold of −10 bits. We subdivided matches by score, binning them into 1-bit intervals, then calculated frequency distribution of the ten different score bins. In parallel, we exhaustively enumerated all words that could match the PWM with a score of −10 or greater, then calculated the likelihood of each of these words under the same background model we used in the motif discovery process. By weighting each word with its background likelihood, we can obtain an expected histogram of PWM match scores. For each score bin, we compared the observed and expected frequencies using a binomial test. We then picked a score cutoff equal to the highest score of the first bin that was not significantly overrepresented relative to the background model (significance defined as *p* ≤ 0.05). In a few cases, the highest-scoring bins were not significantly overrepresented—generally because of a very small total number of matches in these bins—but slightly lower-scoring bins did show overrepresentation. In these cases, we skipped over up to two high-scoring nonoverrepresented bins and set a threshold based on the first nonoverrepresented bin following an overrepresented bin.

### Construction of PWMs from DNase I footprints.

As positive controls for our analyses, we generated a set of PWMs of the binding specificity for developmentally regulated transcription factors characterized by DNase I footprinting. Footprint data was obtained from version 2.0 of the FlyReg database [9]. To ensure that we observed a representative sampling of binding sites for each factor, we concentrated on the 52 factors with at least five reported footprints. For each of these factors, we extracted genomic sequences for all the footprints plus ten bases of flanking sequence on either side. PWMs were built using NestedMICA [13], requesting a single motif from each set of sequences. Since we expect the concentration of true binding sites in the footprint sequences to be very high, choice of background model is less important than when learning motifs from bulk sequence, so we used a simple, uniform zeroth order background model.

Since we did not know the size of each factor's binding site, we used a similar procedure to that described under the Motif refinement and trimming section. For each factor, we performed several runs with motif lengths between four and 12. We then plotted the evidence for each motif length. In 30 of 52 cases, we saw a clear peak in the evidence plot, and used the corresponding weight matrix as the optimal model for that factor. For the remaining 22 cases, there was not an obvious peak, so we were not able to confidently choose a single model. This may be due to insufficient data (supported by the observation that the factors with no clear optimum had a median of 7.5 sites, compared with 18.5 for those with a sharp evidence peak), contamination of the footprint data with some false positives, or because a single PWM is not a good model of the binding specificity for that factor (perhaps because it binds in several different conformations). The 30 optimal PWMs can be found in Figure S2.

### Comparison of PWMs.

We define a divergence measure between two distributions, *P* and *Q,* over alphabet *A* as:

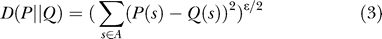
When the exponent, ε, is 1.0, this gives a Cartesian distance. In practice, we found that this gave too much weight to small differences. In this paper, we use an exponent ε = 2.5.

The distance between two PWMs can then be measured as the sum of distances between corresponding positions. Since it is possible that two PWMs could reflect the same motif offset by a few bases in either direction, we consider each motif to be flanked (to infinity in both directions) by uniform distributions over *A,* then consider all possible ungapped alignments between two motifs. The lowest score (i.e*.*, the best alignment) is reported.

We note that there is not an obviously principled way to compare PWMs. This is an ad hoc distance metric selected on the basis that pairs of motifs with low divergences under this metric tended to look similar by eye. Other possible distance metrics include Cartesian distances with a different exponent, KL divergence, and correlation coefficient. These have all been tested but were judged to be inferior to this measure. Better methods for motif comparison, and also techniques for evaluating motif comparison methods, remain an interesting topic for future research.

This comparison measure—along with the ability to identify best matches and reciprocal best matches between two sets of motifs—has been integrated into the MotifExplorer tool, which can be downloaded from http://www.sanger.ac.uk/Software/analysis/nmica/mxt.shtml.

Where a measure of the significance of a match between two motifs is required, we repeatedly shuffle the columns of the query PWM and compare the resulting random motif with the same target motif database. An empirical *p*-value for the significance of the initial match can be obtained by counting the number of times a random motif matches with a score less than or equal to the best match of the query motif.

### Availability.

Source code for the NestedMICA motif-discovery system is freely available under the GNU Lesser General Public License (LGPL) from http://www.sanger.ac.uk/Software/analysis/nmica.

The motif PWMs and annotation can be downloaded from the Tiffin database of computational motif finding results http://servlet.sanger.ac.uk/tiffin.

## Supporting Information

Figure S1 Summary of 120 Motifs Inferred from D. melanogaster 5′ Flanking Sequences Score cutoffs are recommended values based on the protocol described under Materials and Methods. Matches upstream of *Drosophila* genes indicate the number of times the PWM matched (at the recommended score cutoff) in a set of 200-bp sequences upstream of all annotated genes on release 4 of the D. melanogaster genome. A/T bias is the average fraction of A and T nucleotides in words matching the PWM. Upstream bias? indicates whether the motif is significantly overrepresented immediately upstream of genes on D. melanogaster chromosome arm 2L. Conserved? indicates whether high-scoring matches of this weight matrix to the genome are preferentially conserved in other drosopholids, as described in the text. Forward orientation shows the fraction of the PWM matches to upstream sequences in a forward orientation relative to the direction of transcription, and Orientation bias is a binomial *p*-value indicating whether the fraction of forward-oriented motifs deviates significantly from the expected 50%.
(811 KB PDF)

Figure S2 30 Motifs Inferred from DNAse I Footprints (134 KB PDF)

Figure S3 172 Motifs from JASPAR CORE and Additional SELEX Evidence (534 KB PDF)

Table S1 Statistics of 120 Motifs Inferred from D. melanogaster 5′ Flanking Sequences This table contains the same data as Figure S1, provided in a format that is more suitable for further analysis.
(5 KB TDS)

## Abbreviations

TFBM: transcription factor binding motif
TFBS: transcription factor binding sites
