# Supplementary material for: Large-Scale Discovery of Promoter Motifs in Drosophila melanogaster
Source: PLoS Comput Biol. 2007 Jan 19;3(1):e7. doi: 10.1371/journal.pcbi.0030007 (PMC1779301; doi:10.1371/journal.pcbi.0030007)
Supplement: Supplementary file 1 [file pcbi.0030007.sg001.pdf]

Motif	Score cutoff (bits)	Matches upstream of Drosophila genes	A/T bias	Upstream bias?	Conserved?	Forward orientation (%)	Orientation bias (p-value)
TIFDMEM0000001	-0.0	2	0.54	No	No	100.0	0.250
TIFDMEM0000002	-2.0	532	0.79	Yes	No	50.6	0.414
TIFDMEM0000003	-3.0	272	0.51	No	Yes	45.6	0.0815
TIFDMEM0000004	-5.0	1975	0.82	Yes	Yes	50.2	0.428
TIFDMEM0000005	-8.0	1506	0.61	Yes	Yes	51.3	0.158
TIFDMEM0000006	-2.0	424	0.61	Yes	No	51.9	0.233
TIFDMEM0000007	-2.0	21	0.42	No	Yes	28.6	0.0392
TIFDMEM0000008	-5.0	865	0.61	No	Yes	47.2	0.0513
TIFDMEM0000009	-1.0	435	0.46	No	No	34.9	**1.70e-10**
TIFDMEM0000010	-4.0	94	0.39	No	Yes	53.2	0.303
TIFDMEM0000011	-4.0	1567	0.67	Yes	No	50.1	0.480
TIFDMEM0000012	-3.0	1692	0.54	Yes	No	48.9	0.198
TIFDMEM0000013	-2.0	331	0.75	Yes	Yes	48.6	0.330
TIFDMEM0000014	-4.0	465	0.75	Yes	No	52.7	0.133
TIFDMEM0000015	-5.0	992	0.46	Yes	Yes	54.0	**0.00605**
TIFDMEM0000016	-2.0	109	0.70	Yes	Yes	42.2	0.0625
TIFDMEM0000017	-1.0	1798	0.81	Yes	Yes	50.0	0.510
TIFDMEM0000018	-2.0	1954	0.56	Yes	No	47.3	**0.00989**
TIFDMEM0000019	-2.0	2371	0.76	Yes	No	48.8	0.134
TIFDMEM0000020	-3.0	269	0.43	No	No	52.8	0.197
TIFDMEM0000021	-6.0	1709	0.83	Yes	No	48.3	0.0804
TIFDMEM0000022	-6.0	1267	0.68	Yes	No	50.1	0.478
TIFDMEM0000023	-3.0	567	0.60	No	Yes	50.6	0.401
TIFDMEM0000024	-6.0	1785	0.80	Yes	Yes	47.1	**0.00787**
TIFDMEM0000025	-2.0	207	0.58	No	Yes	53.1	0.202
TIFDMEM0000026	-6.0	745	0.44	Yes	Yes	58.0	**7.39e-06**
TIFDMEM0000027	-2.0	55	0.68	No	No	56.4	0.209
TIFDMEM0000028	-2.0	504	0.51	No	Yes	53.0	0.0982
TIFDMEM0000029	-7.0	1928	0.65	Yes	No	53.6	**0.000901**
TIFDMEM0000030	-4.0	800	0.76	Yes	No	47.1	0.0558
TIFDMEM0000031	-5.0	819	0.75	No	No	51.0	0.288
TIFDMEM0000032	-2.0	175	0.74	No	No	78.3	**1.23e-14**
TIFDMEM0000033	-3.0	635	0.47	Yes	Yes	52.1	0.151
TIFDMEM0000034	-7.0	4007	0.81	No	Yes	50.6	0.224
TIFDMEM0000035	-3.0	439	0.66	No	No	46.9	0.107
TIFDMEM0000036	-4.0	986	0.45	No	No	57.2	**3.44e-06**
TIFDMEM0000037	-2.0	143	0.54	No	No	65.0	**0.000203**
TIFDMEM0000038	-6.0	2853	0.69	Yes	Yes	50.3	0.397
TIFDMEM0000039	-3.0	609	0.77	Yes	No	48.1	0.186
TIFDMEM0000040	-4.0	1585	0.55	Yes	Yes	54.1	**0.000544**
TIFDMEM0000041	-4.0	808	0.67	Yes	Yes	45.0	**0.00271**
TIFDMEM0000042	-5.0	1355	0.46	Yes	Yes	63.8	**7.59e-25**
TIFDMEM0000043	-0.0	0	0.59	No	Yes	-	-
TIFDMEM0000044	-6.0	561	0.52	Yes	Yes	48.1	0.199
TIFDMEM0000045	-7.0	1348	0.51	Yes	Yes	51.4	0.157
TIFDMEM0000046	-1.0	45	0.61	No	Yes	55.6	0.276
TIFDMEM0000047	-3.0	554	0.74	Yes	No	50.4	0.449
TIFDMEM0000048	-5.0	387	0.71	Yes	Yes	51.2	0.342
TIFDMEM0000049	-6.0	1338	0.73	Yes	No	54.7	**0.000314**
TIFDMEM0000050	-1.0	336	0.53	No	Yes	51.2	0.351
TIFDMEM0000051	-7.0	1728	0.69	Yes	Yes	49.7	0.396
TIFDMEM0000052	-4.0	502	0.69	Yes	Yes	49.8	0.482
TIFDMEM0000053	-2.0	375	0.61	No	Yes	49.6	0.459
TIFDMEM0000054	-3.0	686	0.79	Yes	Yes	52.8	0.0789
TIFDMEM0000055	-4.0	1347	0.65	Yes	Yes	49.4	0.351
TIFDMEM0000056	-1.0	232	0.67	Yes	Yes	49.1	0.422
TIFDMEM0000057	-3.0	190	0.30	Yes	Yes	53.7	0.173
TIFDMEM0000058	-2.0	285	0.40	No	Yes	49.8	0.500
TIFDMEM0000059	-1.0	313	0.51	No	No	54.0	0.0874
TIFDMEM0000060	-1.0	23	0.61	No	Yes	21.7	**0.00531**
TIFDMEM0000061	-1.0	1109	0.78	Yes	No	54.6	**0.00109**
TIFDMEM0000062	-4.0	692	0.71	No	No	47.3	0.0798
TIFDMEM0000063	-4.0	1114	0.40	No	Yes	50.1	0.488
TIFDMEM0000064	-5.0	3127	0.73	Yes	Yes	48.5	0.0464
TIFDMEM0000065	-2.0	351	0.57	No	Yes	54.7	0.0437
TIFDMEM0000066	-1.0	767	0.77	Yes	No	50.2	0.471
TIFDMEM0000067	-4.0	1009	0.56	Yes	Yes	48.9	0.244
TIFDMEM0000068	-3.0	1730	0.87	No	Yes	50.4	0.377
TIFDMEM0000069	-4.0	743	0.55	No	No	48.6	0.232
TIFDMEM0000070	-5.0	2934	0.79	Yes	Yes	52.0	0.0169
TIFDMEM0000071	-2.0	85	0.52	No	Yes	44.7	0.193
TIFDMEM0000072	-3.0	2662	0.71	Yes	Yes	52.0	0.0190
TIFDMEM0000073	-3.0	728	0.65	Yes	Yes	49.0	0.315
TIFDMEM0000074	-6.0	1258	0.55	Yes	Yes	51.0	0.259
TIFDMEM0000075	-5.0	1869	0.77	Yes	No	50.1	0.463
TIFDMEM0000076	-6.0	1365	0.29	No	Yes	51.8	0.0969
TIFDMEM0000077	-1.0	16	0.65	No	Yes	81.3	0.0106
TIFDMEM0000078	-3.0	479	0.75	Yes	Yes	45.1	0.0177
TIFDMEM0000079	-1.0	612	0.48	Yes	Yes	50.0	0.516
TIFDMEM0000080	-5.0	797	0.68	No	Yes	49.8	0.472
TIFDMEM0000081	-1.0	28	0.64	No	Yes	35.7	0.0925
TIFDMEM0000082	-2.0	358	0.58	No	Yes	53.4	0.112
TIFDMEM0000083	-5.0	876	0.71	Yes	Yes	70.4	**9.67e-35**
TIFDMEM0000084	-2.0	338	0.73	Yes	Yes	50.9	0.393
TIFDMEM0000085	-6.0	8267	0.79	Yes	Yes	51.2	0.0173
TIFDMEM0000086	-4.0	789	0.80	Yes	No	50.8	0.335
TIFDMEM0000087	-1.0	86	0.51	No	Yes	50.0	0.543
TIFDMEM0000088	-7.0	1977	0.46	No	Yes	47.7	0.0239
TIFDMEM0000089	-4.0	401	0.64	No	No	49.1	0.382
TIFDMEM0000090	-1.0	2048	0.63	Yes	No	49.0	0.182
TIFDMEM0000091	-7.0	1110	0.72	Yes	Yes	64.1	**2.81e-21**
TIFDMEM0000092	-4.0	407	0.69	No	No	50.4	0.461
TIFDMEM0000093	-2.0	1223	0.63	Yes	Yes	46.9	0.0172
TIFDMEM0000094	-1.0	127	0.76	No	Yes	58.3	0.0378
TIFDMEM0000095	-1.0	204	0.84	No	No	56.9	0.0292
TIFDMEM0000096	-5.0	989	0.64	Yes	No	48.6	0.204
TIFDMEM0000097	-2.0	643	0.60	Yes	No	48.2	0.193
TIFDMEM0000098	-7.0	2041	0.64	Yes	Yes	48.0	0.0347
TIFDMEM0000099	-4.0	209	0.37	No	Yes	51.2	0.391
TIFDMEM0000100	-3.0	717	0.61	No	No	49.0	0.301
TIFDMEM0000101	-3.0	711	0.36	No	Yes	45.0	**0.00431**
TIFDMEM0000102	-3.0	71	0.35	Yes	Yes	46.5	0.318
TIFDMEM0000103	-1.0	61	0.72	No	Yes	47.5	0.399
TIFDMEM0000104	-6.0	1404	0.81	Yes	No	48.1	0.0786
TIFDMEM0000105	-2.0	753	0.69	No	Yes	45.3	**0.00535**
TIFDMEM0000106	-3.0	356	0.71	No	Yes	45.2	0.0401
TIFDMEM0000107	-5.0	2031	0.65	Yes	Yes	67.1	**1.50e-54**
TIFDMEM0000108	-3.0	1079	0.85	Yes	Yes	48.0	0.100
TIFDMEM0000109	-5.0	552	0.52	Yes	Yes	43.3	**0.000932**
TIFDMEM0000110	-1.0	73	0.74	No	Yes	38.4	0.0302
TIFDMEM0000111	-6.0	2105	0.75	Yes	No	46.6	**0.000846**
TIFDMEM0000112	-4.0	1482	0.54	Yes	Yes	50.7	0.311
TIFDMEM0000113	-2.0	431	0.80	Yes	No	49.9	0.500
TIFDMEM0000114	-5.0	2591	0.69	Yes	No	49.4	0.278
TIFDMEM0000115	-1.0	262	0.52	Yes	Yes	50.0	0.525
TIFDMEM0000116	-7.0	641	0.40	Yes	Yes	60.8	**2.23e-08**
TIFDMEM0000117	-6.0	1534	0.47	No	Yes	51.6	0.105
TIFDMEM0000118	-3.0	890	0.62	Yes	Yes	49.6	0.407
TIFDMEM0000119	-5.0	668	0.69	Yes	No	50.0	0.515
TIFDMEM0000120	-5.0	1273	0.54	No	Yes	50.4	0.411
