# Supplementary material for: Large-Scale Discovery of Promoter Motifs in Drosophila melanogaster
Source: PLoS Comput Biol. 2007 Jan 19;3(1):e7. doi: 10.1371/journal.pcbi.0030007 (PMC1779301; doi:10.1371/journal.pcbi.0030007)
Supplement: Supplementary file 2 [file pcbi.0030007.sg002.pdf]

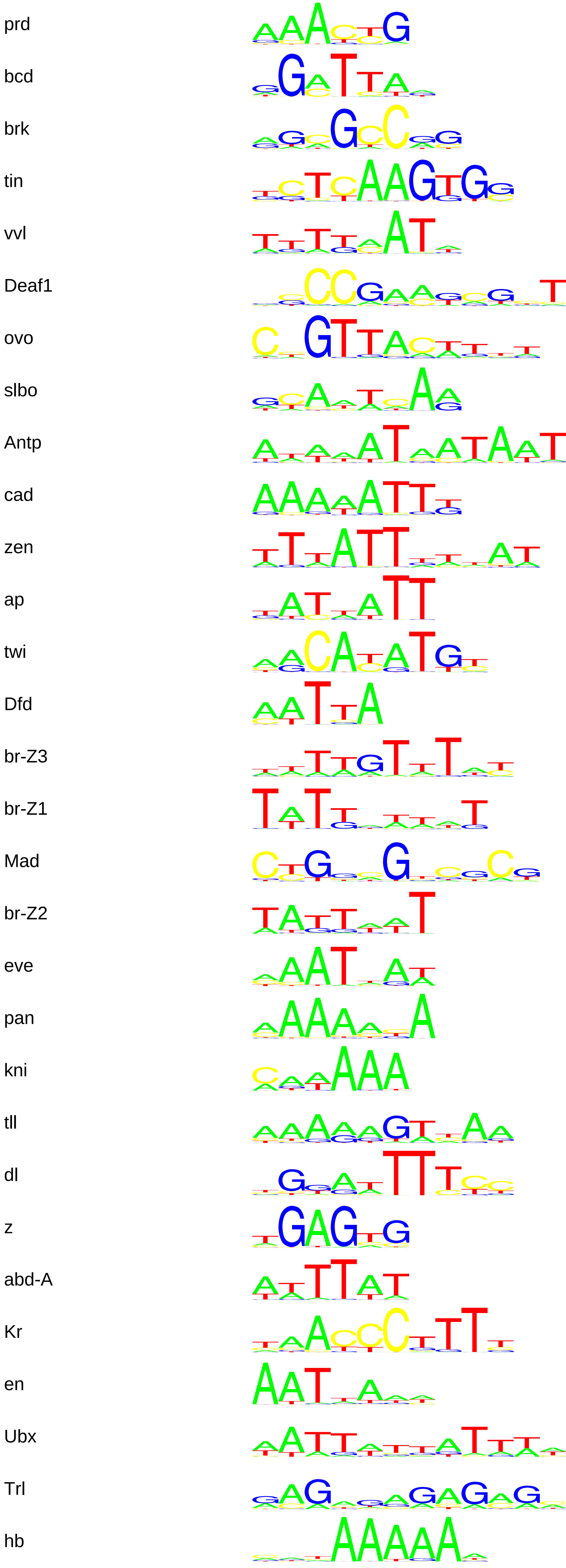
prd
bcd
brk
tin
vvl
Deaf1
ovo
slbo
Antp
cad
zen
ap
twi
Dfd
br-Z3
br-Z1
Mad
br-Z2
eve
pan
kni
tll
dl
z
abd-A
Kr
en
Ubx
Trl
hb
