# Supplementary material for: Large-Scale Discovery of Promoter Motifs in Drosophila melanogaster
Source: PLoS Comput Biol. 2007 Jan 19;3(1):e7. doi: 10.1371/journal.pcbi.0030007 (PMC1779301; doi:10.1371/journal.pcbi.0030007)
Supplement: Supplementary file 3 [file pcbi.0030007.sg003.pdf]

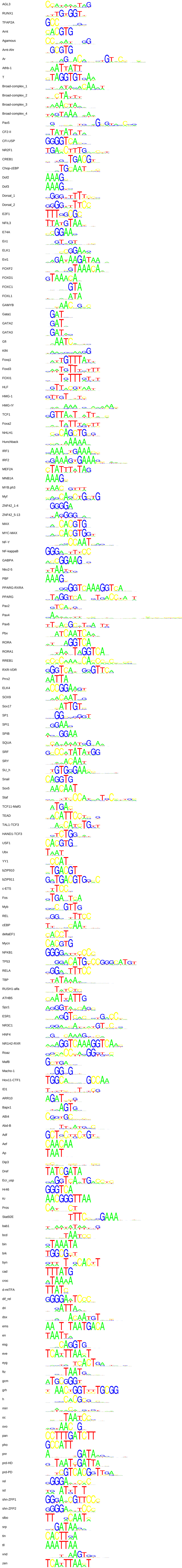

AGL3
RUNX1
TFAP2A
Arnt
Agamous
Arnt-Ahr
Ar
Athb-1
T
Broad-complex_1
Broad-complex_2
Broad-complex_3
Broad-complex_4
Pax5
CF2-II
CFI-USP
NR2F1
CREB1
Chop-cEBP
Dof2
Dof3
Dorsal_1
Dorsal_2
E2F1
NFIL3
E74A
En1
ELK1
Evi1
FOXF2
FOXD1
FOXC1
FOXL1
GAMYB
Gata1
GATA2
GATA3
Gfi
Klf4
Foxq1
Foxd3
FOXI1
HLF
HMG-1
HMG-IY
TCF1
Foxa2
NHLH1
IRF1
IRF2
MEF2A
MNB1A
MYB.ph3
Myf
ZNF42_1-4
ZNF42_5-13
MAX
MYC-MAX
NF-Y
NF-kappaB
GABPA
Nkx2-5
PBF
PPARG-RXRA
PPARG
Pax2
Pax4
Pax6
Pbx
RORA
RORA1
RREB1
RXR-VDR
Prrx2
ELK4
SOX9
Sox17
SP1
SPI1
SPIB
SQUA
SRF
SRY
SU_h
Snail
Sox5
Staf
TCF11-MafG
TEAD
TAL1-TCF3
HAND1-TCF3
USF1
Ubx
YY1
bZIP910
bZIP911
c-ETS
Fos
Myb
REL
cEBP
deltaEF1
Mycn
NFKB1
TP53
RELA
TBP
RUSH1-alfa
ATHB5
Spz1
ESR1
NR3C1
HNF4
NR1H2-RXR
Roaz
MafB
Macho-1
Hox11-CTF1
ID1
ARR10
Bapx1
ABI4
Abd-B
Adf
Aef
Ap
Dip3
Dref
Ecr_usp
Hr46
Kr
Pros
Stat92E
bab1
bcd
bin
brk
byn
cad
croc
d-mtTFA
dif_rel
dri
dsx
ems
en
esg
eve
eyg
ftz
gcm
grh
h
mirr
oc
ovo
pan
pho
pnr
prd-HD
prd-PD
rel
sd
shn-ZFP1
shn-ZFP2
slbo
srp
tin
tll
vnd
zen
